# *“Nutripiatto”*: A tool for nutritional education. A survey to assess dietary habits in preschool children

**DOI:** 10.1371/journal.pone.0282748

**Published:** 2023-03-07

**Authors:** Greta Lattanzi, Claudia Di Rosa, Chiara Spiezia, Roberto Sacco, Samanta Cattafi, Leonardo Romano, Domenico Benvenuto, Silvia Fabris, Laura De Gara, Yeganeh Manon Khazrai

**Affiliations:** 1 Unit of Food Science and Human Nutrition, Campus Bio-Medico University of Rome, Rome, Italy; 2 Unit of Child & Adolescent Neuropsychiatry, Campus Bio-Medico University of Rome, Rome, Italy; 3 Unit of Medical Statistic and Molecular Epidemiology, Campus Bio-Medico University of Rome, Rome, Italy; Università degli Studi di Milano, ITALY

## Abstract

Childhood obesity is a global public health concern linked to metabolic and psychological comorbidities. There is growing evidence that children’s lifestyle habits are shifting towards obesity, with dire consequences for their future well-being and healthcare costs. In this interventional study, we enrolled 115 children aged between 4–5 years (53% females and 47% males) and carried out nutrition education interventions to improve their dietary habits. We introduced “Nutripiatto”, a visual plate icon and easy guide, which was used by the children during the study. We investigated the children’s dietary habits using a Food Frequency Questionnaire at the beginning and end of the study, after one month of using “Nutripiatto”. The results showed that the children significantly increased the portion sizes and frequency of vegetable consumption (P<0.001) and reduced the consumption of several junk foods such as French fries and crisps (P<0.001), reaching the recommended dietary allowances and frequency of consumption. Daily consumption of water also significantly increased, reaching the suggested amount of six glasses per day. Based on these results, “Nutripiatto” can be considered an effective visual guide and helpful tool to achieve small changes and empower families to make healthier food choices. It can also be considered an effective educational tool for nutritionists and healthcare professionals to improve children’s dietary behavior.

## Introduction

Childhood obesity is one of the most significant health challenges of the 21st century [1], as it is rapidly increasing worldwide [2]. According to the World Health Organization (WHO), in 2016, 41 million children under the age of 5, and 340 million children and adolescents aged from 5–19 were found to be overweight or suffering from obesity [2,3]. Data published by the Organisation for Economic Cooperation and Development (OECD) in 2019 revealed that childhood obesity rates are particularly high in Italy [4], ranking fourth worldwide after the United States, New Zealand, and Greece.

The Childhood Obesity Surveillance Initiative (COSI) conducted by "Okkio alla Salute" analyzed the prevalence of overweight and obesity among Italian school children aged between 6–7 years in 2019 and found that 20.4% were overweight and 9.4% [5] were obese. The survey also revealed that 8.7% of children skipped breakfast, while 35.6% consumed a small breakfast, and 55.2% compensated by eating more during mid-morning snacks. Additionally, 48.3% of the children consumed sweet and salty snacks at least three times a week, while legumes were consumed less than once a week by 38.4% of the children interviewed. One in five children did not consume any fruits or vegetables daily, while 25.4% consumed sweet drinks. Many children led a sedentary lifestyle and almost half of them spent more than two hours every day watching TV, playing with tablets, mobile phones, and video games.

It is recommended that obesity prevention and healthy lifestyle education start in early childhood as this is the phase that precedes the adiposity rebound that can lead to obesity [6,7]. Studies have also shown that schools are the best setting for nutrition education, as this is where children spend most of their time. The literature suggests that the best results are achieved when interventions include both diet and physical activity, particularly for preschool children [8,9].

The growing prevalence of childhood obesity is associated with metabolic and psychological comorbidities, with a plethora of diseases that used to be related only to adults such as type 2 diabetes, hypertension, dyslipidemia, etc. [10] with dire consequences for children’s future well-being and also healthcare costs. Obesity is a multifactorial disease caused by genetic, cultural, and environmental factors, which requires multicomponent interventions that take into account lifestyle changes, behavioral strategies, and active parental involvement [11]. Parents are a powerful role model for their children’s eating behaviors [12,13], which they can influence by improving their own eating patterns. Family mealtimes are also crucial, as they expose children to healthier food choices and smaller portion sizes [13]. Recent studies have evaluated the advantages of family cooking to improve and promote healthy eating habits [14].

The dramatic increase in childhood obesity can also be contained through the concerted efforts of both public and private sectors. The Unit of Food Science and Human Nutrition at Campus Bio-Medico University of Rome, Italy, in collaboration with Nestlé Italia, has thus developed “Nutripiatto”, a visual plate icon along with an intuitive explanatory guide to be used as a dietary education tool for children aged between 4–12 years and their families.

The aim of the present pilot study is to evaluate the effectiveness of “Nutripiatto” as a tangible tool for nutritional education of children aged 4–5 years and their caregivers in reducing portion sizes and improving food choices.

## Materials and methods

The present study took place from September 2019 to March 2020. During the first two weeks, 127 preschool children’s parents were recruited to participate in the study according to the following inclusion and exclusion criteria.

1. Inclusion criteria:
  - Both male and female children
  - Children aged 4–5 years.
2. Exclusion criteria:
  - Children affected by eating disorders
  - Children whose parents failed to understand the study’s objectives or did not participate at the explanatory meeting.

Out of 127, 9% (n = 12) of the children was not included in the study as they either refused or did not show up to the explanatory meetings organized at T0. Thus, 115 children were enrolled from 5 preschool classes.

Parents were asked to sign the informed consent and to fill in a Food Frequency Questionnaire (FFQ) to evaluate their children’s dietary habits and physical activity. Afterwards parents, grandparents or other caregivers and children were invited together with teachers and school kitchen staff to attend an explanatory meeting to learn how to use “Nutripiatto” and its guide (T0). The explanatory meetings were held by a nutritionist and organized several times for small groups of people (8 persons), accordingly to the time in which parents, grandparents or other caregivers, teachers and school staff were recruited. To allow parents, grandparents or other caregivers to participate, completion of the questionnaire and the explanatory meetings took place at school entrance or exit times.

During the meeting, the importance of the right amount of nutrients and micronutrients in the diet as well as the proper portion sizes was emphasized. Parents, grandparents or other caregivers were recommended to read the explanatory guide and to try cooking the recipes with their children to improve their food choices and healthy eating habits. They also had the opportunity to meet the nutritionist after the explanatory meeting if they need further information on “Nutripiatto” or issues related to childhood nutrition. About 10% of the parents wanted a further meeting with the nutritionist to better understand the project and what was expected of them. The food education interventions for children were composed by two interactive sessions on healthy dietary habits and lifestyle tailored to the children’s age. They were mainly interactive interventions with games and practical activities to encourage active learning. Children played with food-shaped toys to learn about the main nutrients they contained. They built a Mediterranean diet food pyramid with printable word scrambles, using flash cards or food group colouring sheets. Nutritionists explained and showed children how to use “Nutripiatto” correctly. At the end of each session, children were asked to draw foods and put them in their “Nutripiatto” to evaluate how much they had learned on food composition and healthy eating.

They were invited to bring their “Nutripiatto” at home, while parents were asked to allow their children to use it for their main meals for a month.

At the end of the one-month period (T1), parents were asked to fill in a second FFQ to evaluate changes in their children’s eating habits compared to baseline.

The study was conducted in accordance with the Declaration of Helsinki and approved by the Ethics Committee Campus Bio-Medico University of Rome for studies involving humans (approval code of the study 131/21).

### “Nutripiatto”

“Nutripiatto” is a tool for nutritional education for children aged 4–12 years designed to promote a correct lifestyle and to reduce the incidence of metabolic diseases among children. “Nutripiatto” is a real-size plate that shows the proportions of food groups composing principal meals. Vegetables occupy the half of the dish, while cereals and protein a quarter, respectively. In fact, some easy examples of the respective foods are represented in the “vegetable half part” of the dish and in the two quarters.

**Fig 1:**
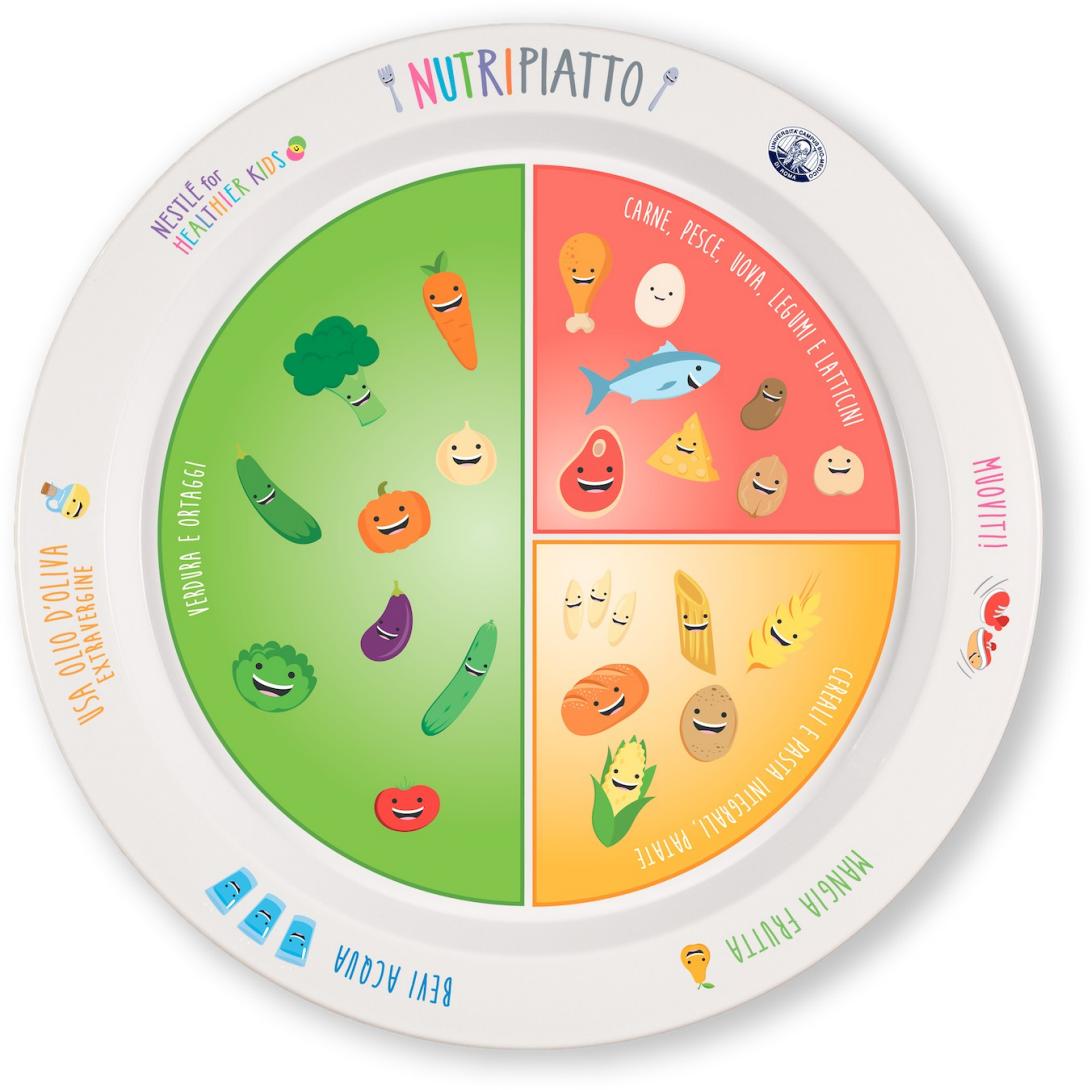
“Nutripiatto”, a visual plate. A tool for nutritional education for children aged 4–12 years.

Healthy eating habits and lifestyle recommendations are also graphically represented on the plate’s edge (Fig 1).

The plate is provided with a booklet that contains information on appropriate portion sizes for children according to their age group as well as easy methods to estimate them. Food portion sizes are illustrated in simple, practical and interactive ways. For instance, children’s hands and household tools were used to estimate portion sizes, accordingly to scientific literature [15,16]. This system proved to be fun for children and useful at the same time, as they could measure portion sizes learning through play.

The guide contains recommendations regarding the importance of hydration, fruit consumption, use of extra virgin olive oil as main seasoning and physical activity.

The booklet contains also recipes (see S1–S4 Figs. for few example of the proposed recipes) for the preparation of healthy meals. Each recipe is designed as a single dish (photographed row and cooked) to estimate the correct portion size and the proportion of food groups to be consumed at each meal in relation to children’s age group. Recipes have been developed according to the nutritional references provided by National Recommended Energy and Nutrient Intake Levels (LARN) [17] for the respective age groups taken into consideration in the booklet (4–6 /7-9/ 10–12 years). Breakfast and snacks provided 15% and 5–10% of the daily energy requirement, respectively, while the principal meals provided about 30–40% of the total daily energy amount with a protein intake less than 15%.

The booklet provided with **“Nutripiatto”** is based on the National Nutritional Guidelines (NNG). It aims to suggest adequate portion sizes and weekly food consumption frequencies, to improve children’s eating patterns and to prevent metabolic diseases [18–20]. A recommended serving size indicates the amount of food that should be consumed during a meal or a snack, while weekly consumption frequencies imply the number of times that a food or a food group should be consumed in a week.

Table 1 shows the three portion sizes for the different food categories: small (for children aged 4–6 years), medium (for children aged 7–9 years), and large (for children aged 10–12 years) according to NNG [18–20].

**Table 1:** Portion sizes. Different portion sizes for each food categories for children aged 4–6 years, 7–9 years and 10–12 years according to NNG.

FOOD CATEGORIES	SMALL PORTION SIZE (age 4–6 yrs)	MEDIUM PORTION SIZE (age 7–9 yrs)	LARGE PORZION SIZE (age 10–12 yrs)
**CARBOHYDRATES SOURCE**			
Pasta/rice/barley/couscous	50 g	60 g	90 g
Bread	60 g	70 g	110 g
Potatoes	200 g	230 g	360 g
Pizza	150 g	200 g	350 g
Biscuits/Breakfast cereals	30 g (3 biscuits)/30 g	40 g (4 biscuits)/40 g	40 g (4 biscuits)/40 g
**PROTEIN SOURCES**			
Meat*	70 g	80 g	100 g
Fish**	110 g	130 g	160 g
Egg	50 g (n. 1 egg)	50 g (n. 1 egg)	100 g (n. 2 egg)
Legumes	60 g (fresh) and 20 g (dried)	90 g (fresh) and 30 g (dried)	120 g (fresh) and 40 g (dried)
**MILK AND DAIRY PRODUCTS**			
Fresh cheeses/seasoned cheeses	40 g /20 g	70 g/30 g	100 g/50 g
Milk	200 ml	200 ml	200 ml
Yogurt	125 g	125 g	125 g
**VEGETABLES AND FRUIT**			
Vegetables	40 g (raw vegetables) and 120 g (cooked vegetables)	50 g (raw vegetables) and 150 g (cooked vegetables)	50 g (raw vegetables) and 200 g (cooked vegetables)
Fruit	100 g	150 g	200 g
Nuts	20 g	30 g	30 g
**EXTRA VIRGIN OLIVE OIL**	10 g	10 g	10 g
**SWEETS, CONFECTIONARY, AND SNACKS**	30 g baked desserts (donuts, tarts) or 10 g chocolate or jam or 100 g ice cream	50 g baked desserts (donuts, tarts) or 25 g chocolate or jam or 100 g ice cream	100 g baked desserts (donuts, tarts) or 40 g chocolate or jam or 120 g ice cream or 5 g sugar***
**WATER**	200 ml	200 ml	200 ml

*Prefer lean white meats (chicken, turkey, rabbit etc).

**Limit the consumption of larger fish.

*** Limit your sugar intake to the suggested portion size.

The daily and weekly frequencies of consumption of some foods according to the Italian Guideline [18] are reported in Table 2.

**Table 2:** Frequencies of consumption. Daily and weekly frequencies of consumption of some foods according to NNG.

FOOD CATEGORIES	DAILY FREQUENCY OF CONSUMPTION	WEEKLY FREQUENCY OF CONSUMPTION
**CARBOHYDRATES SOURCE**		
Pasta/rice/barley/couscous	2 servings	
Bread	2–3 servings	
Potatoes		1–2 servings
Pizza		1 serving (in place of bread, pasta, rice etc.)
Biscuits/Breakfast cereals	1 serving	
**PROTEIN SOURCE**		
Meat		3 servings
Fish		3–4 servings
Egg		1–2 servings
Legumes		3 servings
**MILK AND DAIRY PRODUCTS**		
Fresh cheeses/seasoned cheeses		2–3 servings
Milk	1 serving	
Yogurt		5–7 servings
**VEGETABLES AND FRUIT**		
Vegetables	2 servings	
Fruit	2–3 servings	
Nuts		3 servings
**EXTRA VIRGIN OLIVE OIL**	3 servings	
**SWEETS, CONFECTIONARY AND SNACKS**	Sugar: 1 serving for elder children	2–4 servings
**WATER**	6 glasses	

### Food Frequency Questionnaire (FFQ)

Two FFQs [21] were used, one at baseline (T0) and one at the end of the study (T1). Both questionnaires were filled in by parents based on their children’s eating patterns and preferences. The FFQs differed from each other because the T1 questionnaire included also a small section about the efficacy of “Nutripiatto”.

The answers to the questionnaire given at T0 and at T1 have been encoded by assigning them a number.

The answers options were different depending on the questions.

#### Section 1

Questions 1,4 and 5: were dichotomous answers “*Yes*” or “*No*” that have been encoded in 1 or 2.

Questions 2 and 3: possible answers were *always* (everyday), *often* (3–4 times/week) *sometimes* (1–2 times/week) and *never* that have been encoded in 1,2,3 and 0 respectively.

#### Section 2 and 3

For food portion sizes the possible answers were *small*, *medium* and *large* that have been encoded in 1, 2 or 3, respectively.

For weekly frequencies of consumption, the possible answers were *always*, *often* (3–4 times/week) *sometimes* (1–2 times/week) and *never* that have been encoded in 1, 2, 3 and 0, respectively.

#### Section 4 (only for T1 questionnaire)

Parents were invited to answer “*Yes*” or “*No*” to the questions that have been encoded in 1 or 2.

The structure of the two FFQs is presented in Table 3.

**Table 3:** Structure of the FFQs. FFQ used at T0 and T1 to evaluate children’s dietary habits and physical activity.

Section	T0 questionnaire	T1 questionnaire
Section 1 (dietary habits and lifestyle)	Does your child follow a specific diet?	
	How many meals do you eat together with your family during the week?	
	Who cooks in your family?	
	Does your child have school lunches?	
	Does your child play any sport?	
Section 2 (food group, portion size and frequency of consumption)	Milk and dairy products (milk, yogurt, cheese…)	
	Breakfast foods (biscuits, toasted bread)	
	Cereals (pasta, rice, bread, pizza) and potatoes	
	Protein foods (legumes, fish, white and red meat, salami, eggs)	
	Fruits and vegetables	
Section 3 (oils and fat, drinks and snacks frequency of consumption)	Oils and animal fats	
	Soft drinks	
	Snacks (sweets, prepackaged snacks, chewing—gum, chocolate)	
Section 4 (Evaluation of *“Nutripiatto”*’s effectiveness)	It was not present	Did you find *“Nutripiatto”* effective as a visual guide?
		Did you notice an increase in the consumption of wholegrain cereals, vegetables and water?
		Did you notice a reduction in the porzion size of animal protein sources?
		Did the level of your child’s physical activity increase?

### Statistical analysis

*GraphPad Prism 9* and *IBM SPSS* were used to perform the analyses.

Data are presented as means ± standard deviation (mean ± DS). Normal distribution of data was checked through the Shapiro-Wilk test.

The non-parametric Wilcoxon signed-rank test was performed to compare the number of children who consumed the right portion size at T1 versus T0. The percentage of children who consumed the medium and large portion sizes and who did not consume some foods at T0 and T1 were compared using the Chi squared test. The level of significance (P-value<0.05) and the effect size (d) (Cohen’s D) were also analysed and their values reported. Daily and weekly frequencies of food consumption were also compared with NNG [18].

## Results

At baseline, 115 children were included in the present study. The majority (94%) were not following a specific diet, except for a small minority, 6% (n = 7), who were on dietary regimes due to being overweight (1%), familial hypercholesterolemia (2%), lactose intolerance (2%), milk protein allergy (1%), and celiac disease (1%). These children thus participated in the nutritional education meetings and received “Nutripiatto” and the booklet, but their data were not included in the analysis in order to prevent bias. The data of 108 children, 53% females (n = 57) and 47% males (n = 51) with a mean age of 4.66±0.48 and 4.57±0.49 respectively, were therefore considered for the analysis. All the children enrolled followed the nutritional program for one month, and at T1, there were no dropouts. Regarding meal timing, 90% of the children’s parents declared that their children consumed breakfast at home, but on working days, they usually had lunch in the school canteen. On the other hand, all children (100%) had dinner at home. Breakfast preparation was equally distributed between parents (52% mothers and 48% fathers), while mothers prepared lunches and dinners on non-working days (78% and 80% respectively). Regarding physical activity, at T0, 82% of children reported that they played sports.

To assess the effectiveness of “Nutripiatto” as a visual guide and helpful tool to improve children’s eating habits, changes in terms of portion sizes and food frequency consumption before and after its use were evaluated.

### Effectiveness of *“Nutripiatto”* on portion sizes

At T1, after one month of using “Nutripiatto”, children showed a greater adherence to portion sizes recommended by NNG [19,20].

Table 4 shows the statistically significant variations in the number of children who consumed the right portion related to the age recommended by NNG before and after using “Nutripiatto”.

**Table 4:** Percentage of children 4–5 yrs who consumed the right portion size before and after the use of “Nutripiatto”.

FOODS	% OF CHILDREN WHO CONSUMED THE RIGHT PORTION SIZE		P VALUE (p)	EFFECT SIZE (d)
	T0	T1		
White pizza (with oil and salt)	43.5	50.0	<0.05	0.2
Red pizza (with tomato sauce)	45.3	53.0	<0.05	0.2
Pasta and rice	17.6	37.0	<0.05	0.6
Soup with pasta and rice	20.4	34.2	<0.05	0.3
Other cereals	21.3	26.0	<0.05	0.5
Bread	50.9	65.7	<0.04	0.2
Roasted potatoes	39	51	<0.05	0.3
French fries	40.7	50	<0.001	0.5
Crisps	39.9	44.5	<0.001	0.5
Biscuits	33.3	46.3	<0.01	0.5
Fish	18.5	64	<0.001	0.8
White and red meat	14.8	63	<0.001	0.9
Vegetables	44.4	23.1	<0.001	-0.6
Eggs	54.6	67.6	<0.003	0.2
Glasses of water	21	51	<0.001	1.56

The statistical analysis was performed using the Wilcoxon signed-rank test. Numerical data were converted into percentages as reported in the Table. P value (p) < 0.05 and effect size (d) > 0.5 indicate values that are statistically significant.

Regarding protein portion sizes, the percentage of children who consumed the right portion of fish increased from 18.5% to 64% (P<0.001; d = 0.8), while the percentage of children who consumed the right portion of white and red meat increased from 14.8% to 63% (P<0.001; d = 0.9).

The percentage of children who consumed the right portion size of French fries and crisps shifted from 40.7% to 50% (P<0.001; d = 0.5) and from 39.9% to 44.5% (P<0.001; d = 0.5), respectively.

A significant increase in the consumption of daily glasses of water was observed at T1, in fact, the percentage of children who drank 6 or more glasses of water per day shifted from 21% at T0 to 51% at T1 (P<0.001; d = 1.56) as suggested by the national guidelines [19,20].

There were no statistically significant variations in the consumption of nuts, milk and dairy products, salami and fruit. Regarding legumes, the right portion sizes were already consumed at T0, thus no significant changes were observed.

The recommended vegetable portion size was the only one that significantly decreased (P<0.001; d = -0.6). This was due to the fact that 76% of children at T1 increased their consumption of vegetables, from the right portion (small) to the medium or large portion sizes. This was probably due to the emphasis given to the importance of consuming vegetables and fibers during the explanatory meetings for both parents and children since vegetable are usually one of the food groups least consumed by children. It is worth noting that children’s fiber intake is lower than those recommended by NNGs throughout Europe, even though it correlates with a lower risk of developing obesity and type 2 diabetes [22]. On the other hand, it is debatable whether a large consumption of dietary fiber could affect the mineral bioavailability [23,24].

Conversely, Table 5 shows the statistically significant variations in children who consumed the medium and large portion sizes after the use of “Nutripiatto”.

**Table 5:** Percentage of children 4–5 yrs who consume the medium and large portion size before and after the use of “Nutripiatto”.

FOODS	% OF CHILDREN WHO CONSUMED THE MEDIUM PORTION SIZE		P VALUE (p)	EFFECT SIZE (d)	% OF CHILDREN WHO CONSUMED THE LARGE PORTION SIZE		P VALUE (p)	EFFECT SIZE (d)
	T0	T1			T0	T1		
Roasted potatoes					6	0	0.01	0.5
Crisps	23	7	0.001	0.6				
Fish	74	32	0.001	1.6				
White and red meat	78	35	0.0001	1.5				
Biscuits					14	4	0.008	0.5
Vegetables	45	65	0.004	0.5				

The statistical analysis was performed using the Chi-squared test. Numerical data were converted into percentages as reported in Table 5. P value (p) < 0.05 and effect size (d) > 0.5 indicate values that were statistically significant.

The reduction in consumption of the medium and large portions of some foods confirms the increase in the consumption of the right portion size for the considered age group (4–5 years).

Only for vegetables did the percentage of children who consumed the medium portion size increase after the use of “Nutripiatto” due to the importance given to vegetable consumption during the class meetings, as previously stated.

Some children did not consume the foods mentioned in the questionnaire. After using “Nutripiatto”, the percentage of children who completely avoided eating specific foods decreased. Of particular importance, the percentage of children who did not consume vegetables decreased from 7% at T0 to 1% at T1 (P<0.01; d = 0.47). A statistically significant decrease in the percentage of children who avoided consuming cereals such as pasta and rice was also observed, shifting from 4% at T0 to 0% at T1 (P<0.001; d = 0.4). Similar results were found for the consumption of biscuits: the percentage of children who did not consume biscuits decreased from 7% at T0 to 1% at T1 (P<0.01; d = 0.4). Conversely, regarding the consumption of other cereals, white and red pizza, roast potatoes, crisps, French fries and nuts, the percentage of children who did not consume the above-mentioned foods increased, although not in a statistically significant way. The consumption of white and red meat, fish and eggs did not undergo any variations after using of “Nutripiatto”.

### Effectiveness of *“Nutripiatto”* on frequency of consumption

The results showed a greater adherence to the recommended frequencies of consumption for children aged 4–5 years according to the NNG [19]. After using “Nutripiatto”, the frequency of consumption of various foods such as biscuits, bread, crisps, fish, soft drinks, etc. became more aligned with the guidelines. For example, biscuit consumption decreased from 5 times per week to 4 times per week (P<0.0006), and bread consumption decreased from 3 times per day to 2 times per day (P<0.009). A significant number of children stopped consuming crisps and soft drinks at T1, compared to consuming them once or "sometimes" per week at T0 (P<0.003 and P<0.001, respectively). Fish consumption increased from once per week to twice per week (P<0.003), and vegetable consumption increased from once a day at T0 to twice a day at T1 (P<0.001) as recommended by NGG [19].

### Effectiveness of *“Nutripiatto”*

Regarding the evaluation of “Nutripiatto” as an effective visual guide, 97% of the children’s parents responded affirmatively. The results also showed that according to the parents, children increased the consumption of wholegrain cereals (23%), consumed more vegetables (61%) and drank more water (62%).

According to 52% of parents, their children reduced their portion sizes of animal protein such as meat and fish.

In addition, 31% of children improved their level of physical activity after the education sessions and the use of “Nutripiatto”.

## Discussion and conclusion

The United States Department of Agriculture (USDA) recommends that nutrition education for pre-schoolers should focus on introducing children to healthy eating habits, appropriate portion sizes, and an increased consumption of whole grains, legumes, vegetables, and fruits. It also suggests that learning should be done in a fun and engaging manner [25]. Our study shows that “Nutripiatto” as an educational tool effectively achieved these objectives in the short term.

As with its predecessors, "My Plate" [26] and "The Healthy Eating Plate" [27], “Nutripiatto” is a visual representation of portion sizes and proportions of different food groups. Our results indicate that when used ap-appropriately, it can be a useful tool for making small changes and empowering families to make healthier food choices [28]. However, there have only been a few intervention studies involving children aimed at evaluating the effectiveness of "My Plate" and "The Healthy Eating Plate" as nutrition education tools. These studies had small sample sizes or high dropout rates. A small study conducted in the U.S. involving children in grades 2–5 showed significant improvements in healthy food choices such as fruits and vegetables compared to the baseline [29]. Another study found no significant differences in food choices between the experimental and control groups, with the exception of increased vegetable and dairy product intake in the intervention group [30]. A study by Metzler et al. (2017) designed a nutrition education curriculum consisting of five 30-minute lessons for kindergarten children based on "My Plate" recommendations. Although children increased their knowledge of healthy eating, there was no evidence of behavioural change. The retention rate was low, with only 52.6% of parents responding to the survey sent home after the intervention [31].

In the present study, there were no dropouts, and all parents answered the FFQ at T1. This could be at-tributed to the fact that the schools strongly supported the intervention and encouraged parents’ participation. The presence of a nutritionist who actively interacted with parents and children was an important factor in increasing the effectiveness of the education intervention. Additionally, compared to previous studies, “Nutripiatto” involved an actual plate that children took home and used for at least one month. This helped parents learn how to properly assess portion sizes and maintain their focus on the educational project. The plate’s colourful design appealed to children, who continued to use it consistently.

Portion sizes have been identified as a risk factor for childhood obesity, as parents tend to overfeed their children [32,33]. Parents can be trained to serve appropriate portion sizes using tools such as food pictures or by preparing food and sharing the experience in group meetings with other parents [34]. As larger portion sizes lead to higher energy intake, it has been suggested to increase the amount of vegetables included in the main meal. Used as an iconic model, “Nutripiatto” embodies these concepts, as it considered portion sizes and the right proportion of food groups, with half of the plate filled with vegetables. The booklet recommends that parents prepare foods with their children to enable them to improve their food choices and learn about portion sizes. By the end of one month, children had used the Nutripiatto’s eye-catching graphics to increase their consumption of vegetables and reduce their intake of junk foods [35].

Childhood obesity is often caused by an imbalance between the calories consumed and expended [36]. It is well-known that we live in an obesogenic environment, which encourages the excessive intake of unhealthy foods and a sedentary lifestyle from an early age [37]. Parents often set poor examples of eating habits and behaviour for their children, such as using smartphones or watching TV while sitting at the dinner table.

Moreover, this type of behaviour reduces parents’ sensitivity and their ability to respond to their children’s needs, which can lead to reduced parent-child interactions [38]. In contrast, regular family meals free from electronic devices such as TVs, mobile phones, etc. should be encouraged because they improve relationships and can be used as appropriate situations to discuss healthy foods [39].

According to the OECD document (2019) [4], barely half of the Italian population, including adults and children, eat a healthy diet in accordance with national guidelines. Less than 40% consume five portions of fruit and vegetables per day [40,41]. Italian dietary guidelines [18] recommend that pre-school and school-age children consume healthy foods such as vegetables, fruits, whole grains and legumes, which are rich in fiber and maintain satiety for longer periods [42,43], and also reduces insulin secretion. Simple sugars, saturated and trans fats should be reduced as they have been associated with increased adiposity and exposure to high LDL- cholesterol levels and hyperglycemia in children [44,45]. In this study, preschool children significantly increased their consumption of vegetables, reaching 21 portions per week, and reduced their consumption of bread and potato chips as snacks.

The results of this study show that “Nutripiatto” can be considered an effective educational tool for improving portion sizes and food choices, particularly when it is combined with food education interventions given by an expert. It can be useful for nutritionists and health professionals to improve the eating behaviour of children and, potentially, the whole family. Although the results are encouraging, the sample size should be expanded together with the period of intervention to assess the long-term effectiveness of this new education tool. Furthermore, it would be interesting to customize nutrition education programs to include other age groups covered by “Nutripiatto”, from 6 to 12 years old.

## Supporting information

S1 Fig “The fake fish”. Row recipe for children aged 4–5.
(TIF)

S2 Fig “The fake fish”. Cooked recipe for children aged 4–5.
(TIF)

S3 Fig “Stuffed tomato”. Row recipe for children aged 4–5.
(TIF)

S4 Fig “Stuffed tomato”. Cooked recipe for children aged 4–5.
(TIF)
