# Correction: *“Nutripiatto”*: A tool for nutritional education. A survey to assess dietary habits in preschool children

**DOI:** 10.1371/journal.pone.0294738

**Published:** 2023-11-15

**Authors:** Greta Lattanzi, Claudia Di Rosa, Chiara Spiezia, Roberto Sacco, Samanta Cattafi, Leonardo Romano, Domenico Benvenuto, Silvia Fabris, Laura De Gara, Yeganeh Manon Khazrai

The Funding and Competing Interests statements are incorrect.

The correct Funding statement is: This article received support from Nestlè Italia, in the forms of the photos included in the article’s figures and the plates that were distributed to the families for nutritional education sessions and to be used at home. Nestlè Italia had no role in study design, data collection and analysis, decision to publish, or preparation of the manuscript. No additional external funding or non-financial support was received for this study.

The correct Competing Interests statement is: The authors have read the journal’s policy and have the following Competing Interests to declare: Nestlè Italia provided non-financial support for this study. This does not alter our adherence to *PLOS ONE* policies on sharing data and materials. There are no patents, marketed products or products in development associated with this research.

## References

[1] Lattanzi G, Di Rosa C, Spiezia C, Sacco R, Cattafi S, Romano L, et al. (2023) “*Nutripiatto*”: A tool for nutritional education. A survey to assess dietary habits in preschool children. PLoS ONE 18(3): e0282748. doi: 10.1371/journal.pone.0282748 36881589 PMC9990952

