# Supplementary figures and images for: “Nutripiatto”: A tool for nutritional education. A survey to assess dietary habits in preschool children
Source: PLoS One. 2023 Mar 7;18(3):e0282748. doi: 10.1371/journal.pone.0282748 (PMC9990952; doi:10.1371/journal.pone.0282748)

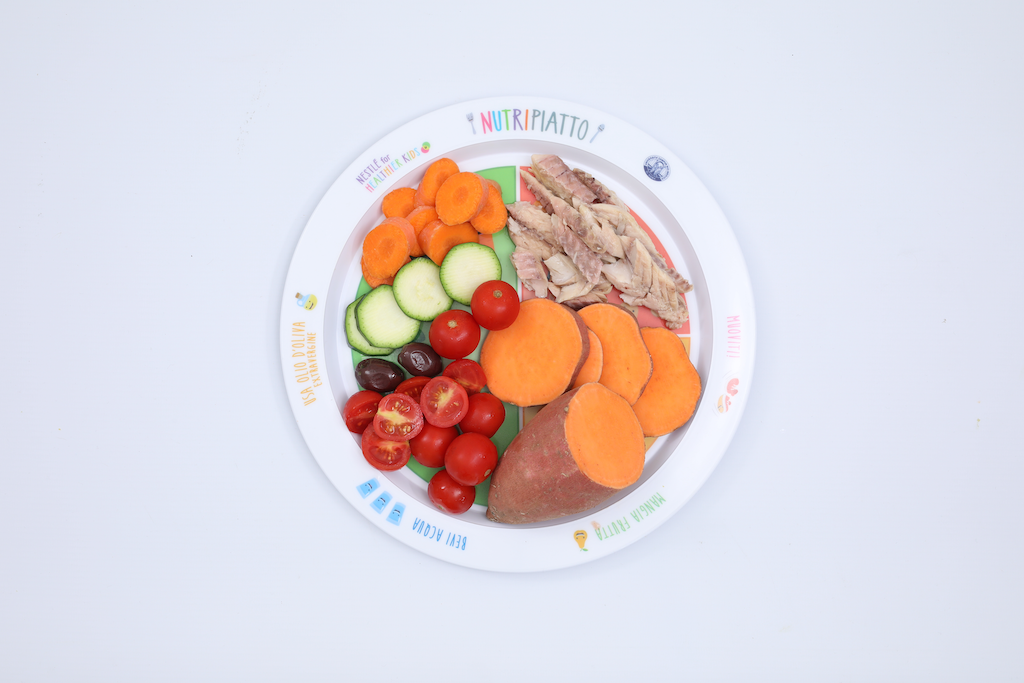

Supplement: S1 Fig — Row recipe for children aged 4–5. (TIF) [file pone.0282748.s001.tif]

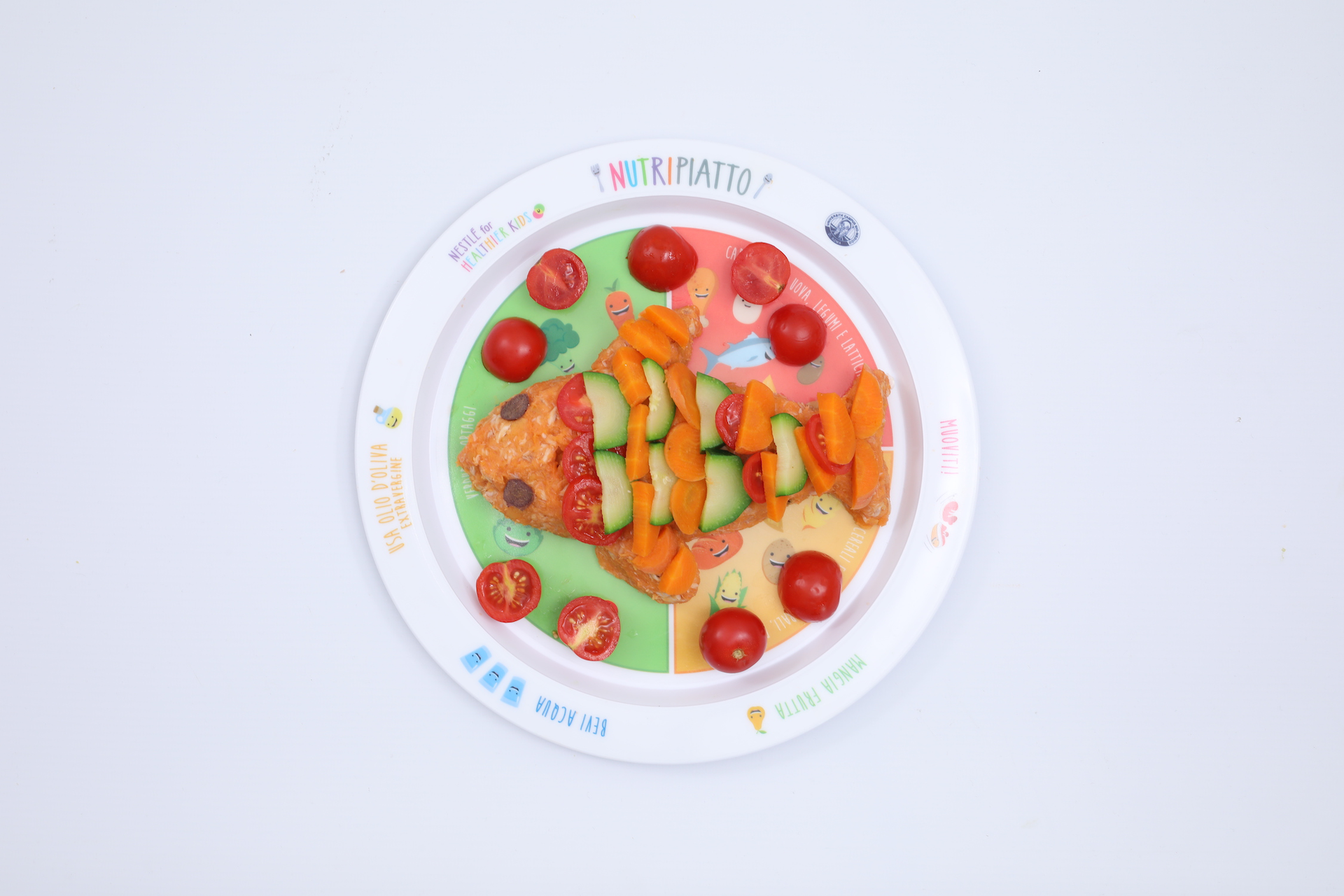

Supplement: S2 Fig — Cooked recipe for children aged 4–5. (TIF) [file pone.0282748.s002.tif]

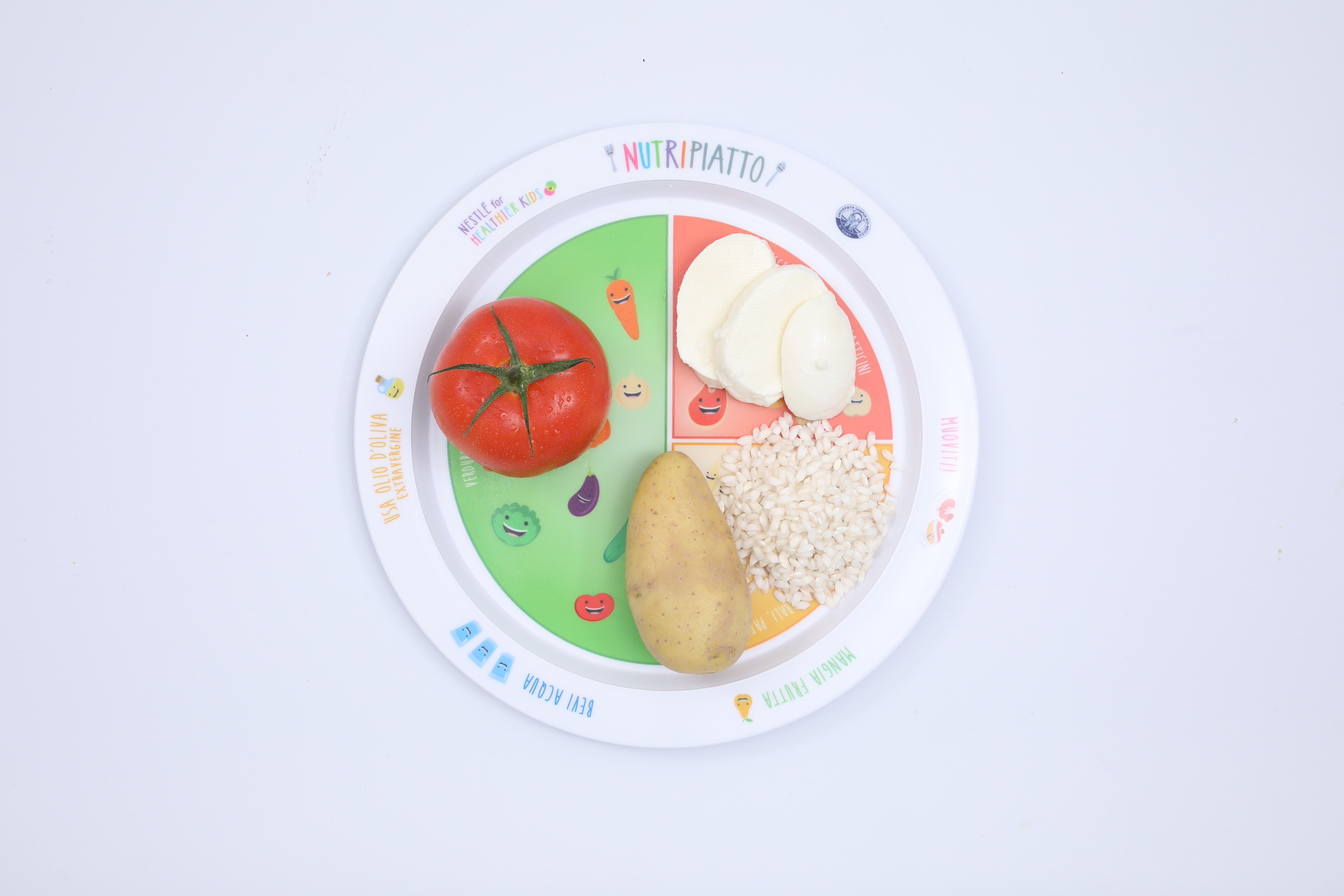

Supplement: S3 Fig — Row recipe for children aged 4–5. (TIF) [file pone.0282748.s003.tif]

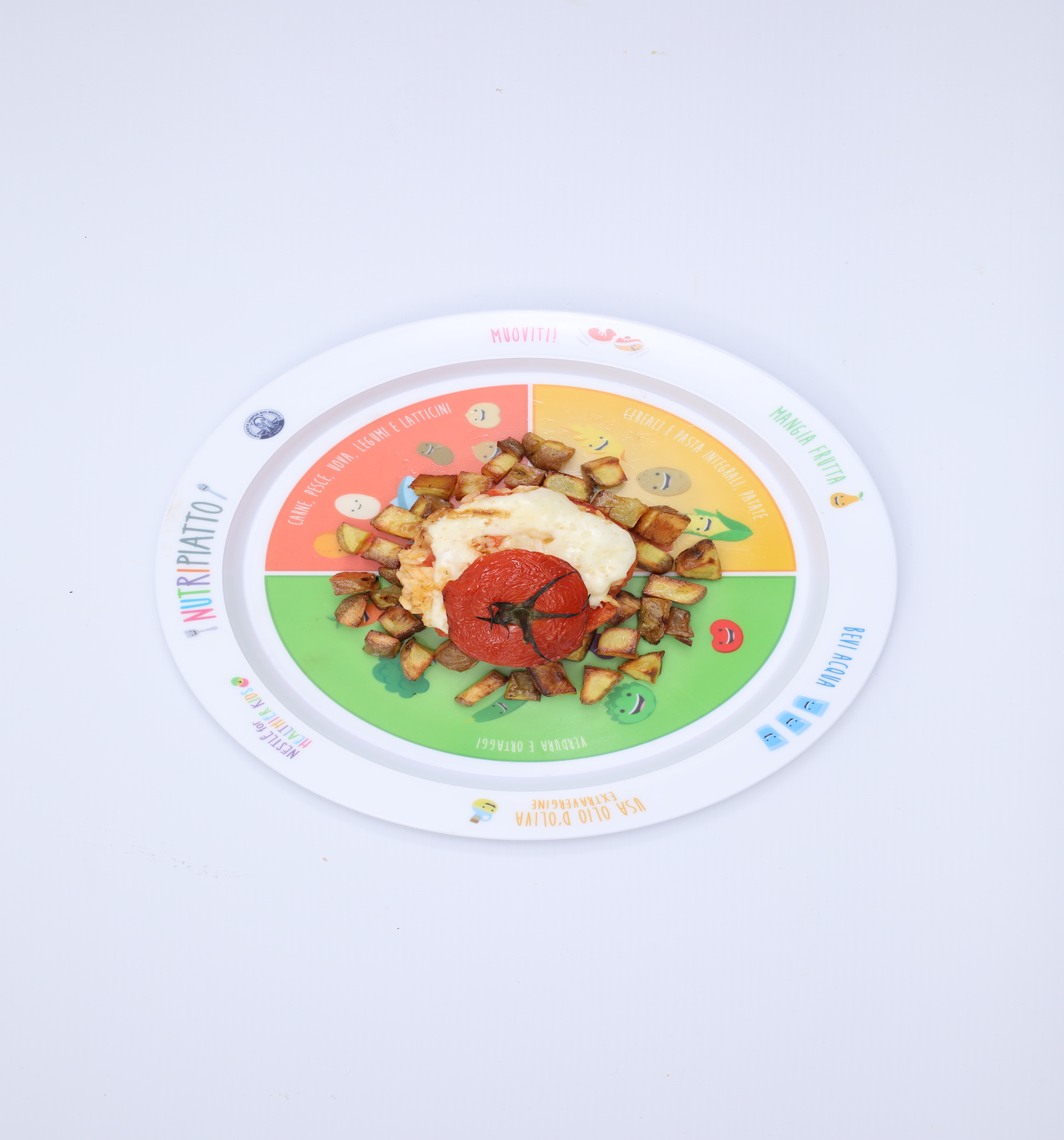

Supplement: S4 Fig — Cooked recipe for children aged 4–5. (TIF) [file pone.0282748.s004.tif]
